# Automatic measurement of lower limb alignment in portable devices based on deep learning for knee osteoarthritis

**DOI:** 10.1186/s13018-024-04658-3

**Published:** 2024-04-10

**Authors:** Jianfeng Yang, Peng Ren, Peng Xin, Yiming Wang, Yonglei Ma, Wei Liu, Yu Wang, Yan Wang, Guoqiang Zhang

**Affiliations:** 1 Department of Orthopedics, the First Medical Center of Chinese PLA General Hospital, Beijing, China; 2 Senior Department of Orthopedics, the Fourth Medical Center of Chinese PLA General Hospital, Beijing, China; 3 Department of Orthopedics, Chinese PLA Southern Theater Command General Hospital, Guangzhou, China; 4 Medical School of Chinese People’s Liberation Army, Beijing, China; 5 Department of Anesthesiology, Guangzhou First People’s Hospital, Guangzhou, China; 6 Damo Academy, Alibaba Group, Hangzhou, China; 7 Department of Orthopedics, the First Medical Center, PLA General Hospital, Fuxing Road, Haidian District, Beijing, China; 8 Department of Orthopedic Surgery, The First Medical Center, Chinese PLA General Hospital, 28 Fuxing Road, Beijing, People’s Republic of China

**Keywords:** Knee osteoarthritis, Deep learning, Lower limb alignment, Total knee arthroplasty

## Abstract

**Background:**

For knee osteoarthritis patients, analyzing alignment of lower limbs is essential for therapy, which is currently measured from standing long-leg radiographs of anteroposterior X-ray (LLR) manually. To address the time wasting, poor reproducibility and inconvenience of use caused by existing methods, we present an automated measurement model in portable devices for assessing knee alignment from LLRs.

**Method:**

We created a model and trained it with 837 conforming LLRs, and tested it using 204 LLRs without duplicates in a portable device. Both manual and model measurements were conducted independently, then we recorded knee alignment parameters such as Hip knee ankle angle (HKA), Joint line convergence angle (JCLA), Anatomical mechanical angle (AMA), mechanical Lateral distal femoral angle (mLDFA), mechanical Medial proximal tibial angle (mMPTA), and the time required. We evaluated the model’s performance compared with manual results in various metrics.

**Result:**

In both the validation and test sets, the average mean radial errors were 2.778 and 2.447 (*P*<0.05). The test results for native knee joints showed that 92.22%, 79.38%, 87.94%, 79.82%, and 80.16% of the joints reached angle deviation<1° for HKA, JCLA, AMA, mLDFA, and mMPTA. Additionally, for joints with prostheses, 90.14%, 93.66%, 86.62%, 83.80%, and 85.92% of the joints reached that. The Chi-square test did not reveal any significant differences between the manual and model measurements in subgroups (*P*>0.05). Furthermore, the Bland-Altman 95% limits of agreement were less than ± 2° for HKA, JCLA, AMA, and mLDFA, and slightly more than ± 2 degrees for mMPTA.

**Conclusion:**

The automatic measurement tool can assess the alignment of lower limbs in portable devices for knee osteoarthritis patients. The results are reliable, reproducible, and time-saving.

## Introduction

Knee osteoarthritis (knee OA), can cause significant joint pain, decreased mobility, and even disability [1]. For patients in the end stage of the condition, Total Knee Arthroplasty (TKA) is a commonly used and highly effective therapy [2]. It is important to properly assess alignment during the perioperative period to ensure optimal outcomes [3]. Lower limb alignment is a critical aspect that surgeons carefully evaluate when assessing deformity, predicting treatment outcomes, and planning for surgery. For cases involving knee OA, the majority of surgeons agree that achieving a neutrally aligned lower limb after TKA is a crucial objective [4].

Long-leg radiographs of anteroposterior X-ray (LLR) quantify alignment by identifying specific anatomic landmarks [5, 6]. Long-leg radiographs of anteroposterior X-ray (LLR) quantify alignment by identifying specific anatomic landmarks [7]. Currently, surgeons measure alignment using standard rulers, digital calipers, or manual measurements in hospital computer systems like PACS (Picture Archiving and Communication System) [8]. LLR is considered the gold standard for radiological imaging of lower limbs in weight-bearing standing positions, providing insight into their mechanics [5, 6]. However, manual methods for measuring the tibial-femoral angle have drawbacks such as being time-consuming, inconvenient, and having low reproducibility of results. Additionally, the reliability of these methods relies on clinical experience. To address these issues, Takahashi et al. [9] proposed a self-developed method that uses digital technology to measure the angle on a radiographic film. This method requires the observer to identify four standardized points using a mouse and calculate the angle. Sled et al. [10] proposed a landmark-based method for assessing Hip knee ankle angle (HKA) using customized software, addressing common issues in similar methods. In clinical practice, measurement accuracy can be influenced by factors such as body position [11–13] (like loading, flexion, or rotation of lower limbs), image quality [7], and reader experience [14–16]. Even with software assistance, deviations caused by hardware facilities and use can also affect the measurement, which leads to inter- and intra-reader variability and unsatisfied reliability [17–19]. In actual use, landmarks selection manually can also be time-consuming.

With the advancements in Artificial Intelligence, Deep Learning-based image analysis is increasingly being utilized in clinical medicine. As the volume of imaging data grows, AI-powered software can provide high-quality assessment outputs in less time and with reduced effort required from surgeons. Simon et al [3] proposed a fully automated deep learning tool based on the LAMA model for knee alignment assessment and limb length measurements. Tack et al [20] proposed a similar fully automated tool for measuring the HKA from LLR based on YOLOv4 And Resnet Landmark regression Algorithm. According to the researchers, their tools based on various models have achieved satisfactory accuracy. But, Tack et al’s tool only identifies the knee as varus or valgus deformity through HKA, and Simon et al’s model cannot assess lower extremity alignment after TKA. Moreover, there is no diagnostic tool for clinical use resulting from most of the current studies.

For this study, we analyzed the average angle measurements of up to 3 orthopedic surgeons for each LLR and compared them to the results generated by a fully automated tool. The tool was specifically trained to provide clinically significant angles of alignment, both with and without the presence of a prosthesis in LLRs.

## Materials and methods

This study was approved by the Ethics Review Board of the First Medical Center of Chinese PLA General Hospital.

We retrospectively reviewed 1041 digital LLR X-rays (from 623 patients) in the dataset of the General Hospital of the People’s Liberation Army. These X-rays were captured using the uDR 780i Pro Fully Automatic Ceiling-mounted DR (UNITED IMAGING, Shanghai, China) between January and December 2021. Each patient may have undergone multiple pre- and post-operative radiographs. All X-rays were performed for clinical or perioperative measurements.

The inclusion criteria were as follows: (1) patient age ≥ 40; (2) knee OA diagnosis established based on the Chinese Guidelines for the Diagnosis and Treatment of Osteoarthritis (2019 edition) [21]; (3) standing LLR X-rays of both lower limbs; (4) for the same patient, over 90 days between examinations; (5) previous total knee arthroplasty history or no surgery history. The exclusion criteria were as follows: (1) non-standard standing LLR X-rays; (2) severe deformity of the femur or tibia; (3) comorbidities of other diseases that may cause severe knee deformities or joint fusion; (4) comorbidities including infectious arthritis or postoperative joint infection; (5) LLRs showing other implants like uni-compartmental knee prosthesis or internal fixation; (6) incorrect posture (such as rotation or flexion of lower limbs); (7) poor image quality.

### Model architecture

We divide the included LLRs into a training set (837), a validation set (101), and a test set (204) according to approximately 80%:10%:20%. For the training set (LLRs were stored with an average size of 2021 × 2021 and a pixel spacing of 0.2 mm), these were calibrated landmarks by orthopaedic specialists, which was implemented in MATLAB (Mathworks, Natick, MA). Then all images were resized to 640 × 320 pixels with the isotropic spacing of 0.79 mm using a bilinear interpolation algorithm and then normalized for model development.

As Liu et al. [22] demonstrated in their study about preoperative planning of total hip arthroplasty, we formulated the alignment measurement to a task including two branches: (1) landmarks detection branch, and (2) edges prediction branch. Each resized LLR was firstly input into the backbone network to extract high-level features for two branches.

**Fig. 1:**
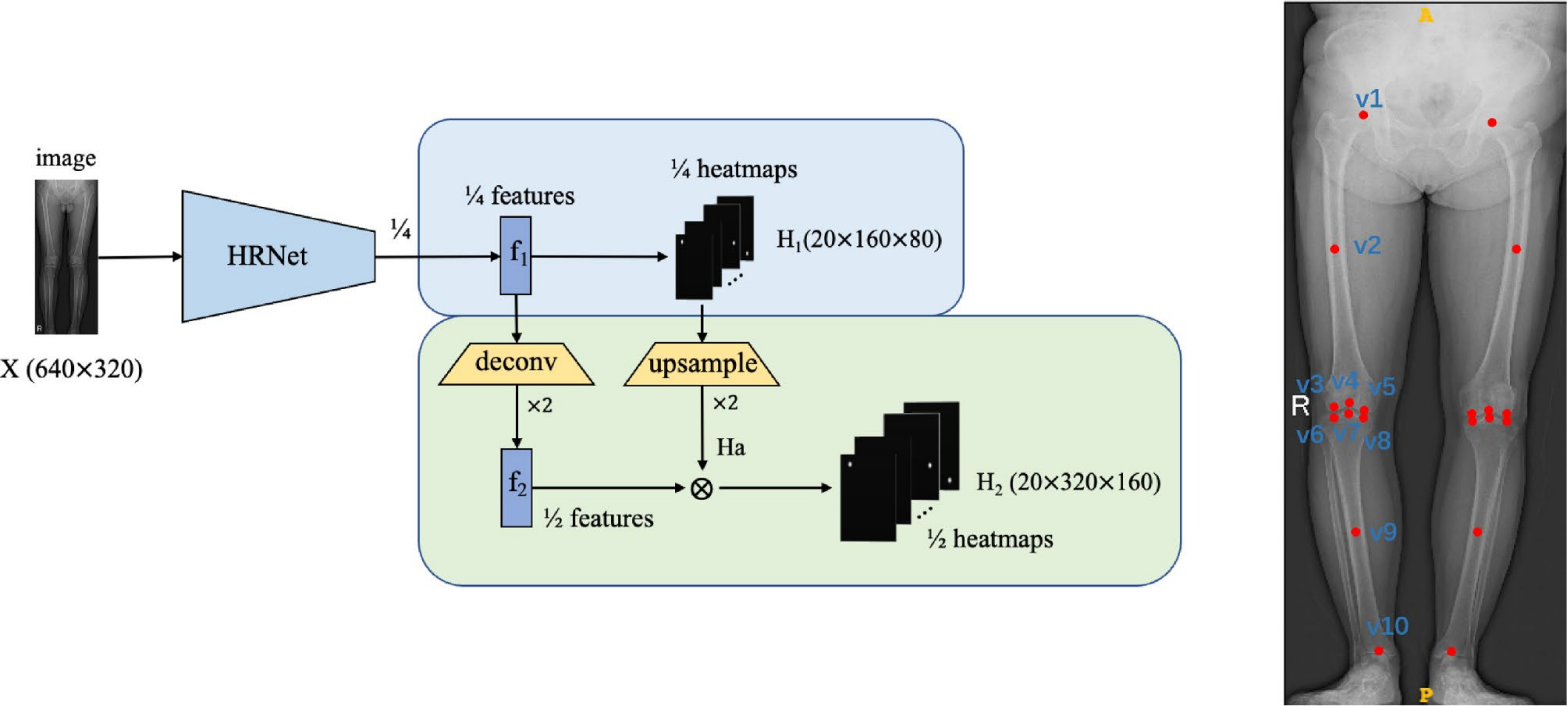
The framework of the proposed method and examples of corresponding landmarks. v1, Centre of the femoral head; v2, Centre of the femoral diaphysis; v3, Lowest point of lateral femoral condyle or prosthesis; v4, Centre of the knee joint or prosthesis on the femoral side; v5, Lowest point of medial femoral condyle or prosthesis; v6, Lowest point of lateral tibial plateau or prosthesis; v7, Centre of the knee joint or prosthesis on the tibia side; v8, Lowest point of medial tibial plateau or prosthesis; v9, Centre of the tibia diaphysis; v10, Centre of the ankle joint. LLR was from a 65-year-old female patient diagnosed with knee OA and authorized consent was obtained from the patient before the use

As for the backbone network, we used the HRNet model for 2 branches above(the proposed model is shown in Fig. 1) [23]. For landmarks detection, each landmark was converted into a heatmap with a 2D Gaussian distribution centered at its coordinates (the hyperparameter σ of Gaussian heatmaps is chosen to 2), and the distribution is normalized to [0,1]. For edge prediction, each edge connecting two landmarks was denoted as a vector and normalized during the experiment for faster convergence, which was a constraint for helping correct the detection deviations implicitly during training.

The proposed model was implemented using PyTorch and ran on a machine with 4 Nvidia P100 GPUs. The parameters of the network were initialized with the pre-trained model from the large public dataset ImageNet [24]. In addition, the training set was augmented by several methods commonly used [25]. According to the study of Adam [26], the backbone network was optimized with an initial learning rate of 1e-3, which was decreased to 1e-4 and 1e-5 at the 120th and 170th epochs. During the model development, the training loss curve normally decreased and stabilized without overfitting. The overall optimization is carried out for 200 epochs with a batch size of 32.

### Surgeons’ evaluation

To test the accuracy of the tool, We used 204 images as a training set from knee OA patients who had not undergone surgery, and those who had undergone unilateral or bilateral total knee replacement surgery (Some patients may have multiple images pre- and post-operatively). There was no duplication of the test set with either the training or validation sets. To assess the measurements of the proposed model, after removing patient information, each LLR was measured by two senior surgeons on a blinded basis independently in PACS, including HKA, JCLA(Joint line convergence angle), AMA(Anatomical mechanical angle), mLDFA(mechanical Lateral distal femoral angle), and mMPTA(mechanical Medial proximal tibial angle). They accomplished this by manually identifying and connecting the corresponding landmarks. The average of the measurements for the landmarks and angles of alignment were considered to be the ground truths. In the portable device, all LLRs were captured using mobile phones (iPhone X, Apple Inc.). During the photo session, the phone was mounted on a tripod, positioned 40 cm away from the LLRs, and centered with them for constant indoor lighting. The images were saved in JPG format and had a resolution of 4000 × 3000 pixels. These images were not optimized or pre-processed before being fed into the model, and the model output the predictions.

### Statistical analysis

To compare the performance of landmarks detection, we adopted mean radial error (MRE) for quantitative comparison, which was defined as

$$MRE=1/n{\sum }_{i=1}^{n}{R}_{i}$$

$n$ denoted the number of detected landmarks and ${R}_{i}$ was the Euclidean distance between the predicted landmarks coordinates obtained by extracting the maxima on heatmaps and the ground truths. To assess the results of the proposed model, we adopted the Chi-Square test. *P* ≥ 0.05 was considered to represent no significant difference between manual measurements and model calculations. Bland-Altman was adopted to assess the consistency between the two methods. For angles, previous studies considered a difference of > 2° as clinically relevant [3], so we adopted >1° and >2° respectively. The data were analyzed using R (4.0.0) and SPSS Version 25.0.0.2 (SPSS, Inc., Chicago, Ill.).

## Result

For comparing the results of the validation and test sets, the average of MREs were 2.778 and 2.447 between the two datasets, and MREs of each landmark were listed in Table 1. For both the model and the clinical use, each side of the lower limb was measured separately. In the test set, 204 images from 167 patients were included (the demographics and morphological data were shown in Table 2), 98.09% (257 of 262) of those with a native knee joint were identified, and 97.26% (142 of 146) of those with a prosthesis identified (as shown in Table 3).

**Table 1:** The MRE for landmark and average in the validation and test sets

Landmarks Datasets	V1	V2	V3	V4	V5	V6	V7	V8	V9	V10	mean
**Validation set**	5.432	4.429	2.266	1.514	2.226	2.547	2.081	2.325	3.223	1.740	2.778
**Test set**	4.131	2.978	2.272	1.508	2.233	2.395	2.012	2.309	3.105	1.528	2.447

**Table 2:** Demographics and morphological data in test set

Variables	Test set (*n* = 408 knee joints)
	n (%) or Mean ± SD (Range)
**Native Knee Joint**	262
number of detected†	257 (98.1%)
number of undetected†	5 (1.9%)
**sex(female/male)**	115/39
age(year)*	62.72 ± 8.16
BMI*	25.98 ± 3.33
**side(left/right)**†	133 (51.8%)/124 (48.2%)
**K-L grade**†	
K-L 2	25 (9.7%)
K-L 3	107 (41.6%)
K-L 4	125 (48.6)
**length of symptoms**†	
<5 years	58 (22.6%)
5–10 years	119 (46.3%)
>10 years	80 (31.1%)
**alignment status**†	
varus	140 (54.5%)
neutral	79 (30.7%)
valgus	38 (14.8%)
**Joint with Prothesis**	146
number of detected†	142 (97.3%)
number of undetected†	4 (2.7%)
**sex(female/male)**	73/29
age(year)*	65.33 ± 5.11
BMI*	27.32 ± 3.47
**side(left/right)**†	63 (44.4%)/79 (55.6%)

*the values are given as the mean and standard deviation;

†the values are given as the number with the percentage in parentheses

**Table 3:** Correlations and agreement of manual and automatic measurements of alignment and used time

	Knee Joint†	MAD*	% AD<1°†	% AD<2°†	P value
**Native Joint**					
HKA	257	0.47 ± 0.38	92.22%	99.20%	
JCLA	257	0.65 ± 0.60	79.38%	96.50%	
AMA	257	0.48 ± 0.44	87.94%	99.22%	
mMPTA	257	0.79 ± 0.87	79.82%	93.39%	
mLDFA	257	0.62 ± 0.54	80.16%	97.67%	
Used time*					<0.01
mean manual		173.78 ± 12.37			
model		2.53 ± 0.80			
**Joint with Prothesis**					
HKA	142	0.51 ± 0.40	90.14%	98.59%	
JCLA	142	0.34 ± 0.38	93.66%	98.59%	
AMA	142	0.50 ± 0.43	86.62%	99.30%	
mMPTA	142	0.55 ± 0.46	83.80%	98.59%	
mLDFA	142	0.54 ± 0.42	85.92%	99.30%	
Used time*					<0.01
mean manual		175.04 ± 13.11			
model		2.54 ± 0.80			

MAD, mean absolute difference

AD, average deviation

*the values are given as the mean and standard deviation;

†the values are given as the number with the percentage in parentheses

The automatic measurement results were highly correlated with the standard reference [27]. with the mean absolute difference (MAD) of HKA, JCLA, AMA, mMPTA, mLDFA, being, respectively, 0.47 ± 0.38, 0.65 ± 0.60, 0.48 ± 0.44, 0.79 ± 0.87, 0.62 ± 0.54 for the lower limbs with a native knee joint and 0.51 ± 0.40, 0.34 ± 0.38, 0.50 ± 0.43, 0.55 ± 0.46, 0.54 ± 0.42 for lower limbs with a prosthesis. And the percentage of angle deviation (AD) <1° of HKA, JCLA, AMA, mMPTA, mLDFA, achieved 92.22%, 79.38%, 87.94%, 79.82%, 80.16% for those with a native knee joint and 90.14%, 93.66%, 86.62%, 83.80%, 85.92% for those with a prosthesis respectively. Automated measurement saved over 98% of the time compared to manual methods.

In order to assess whether the measurements were affected by patient characteristics, we classified the test set according to alignment status, sex, BMI, and age (as shown in Table 4). The accuracy was defined as AD<1°. And after we regrouping the patients in the test set according to those, The Chi-square test for HKA showed no significant differences between the model and manual measures in subgroups (*P*>0.05 for each subgroup).

**Table 4:** Accuracy/inaccuracy and Chi-square test of HKA in test set

	Native Joint					Joint with Prothesis			
	accuracy	inaccuracy	χ²	p value		accuracy	inaccuracy	χ²	p value
**Alignment status**									
Varus (≤-2°)	179	14	0.975	0.614		72	6	3.511	0.173
Neutural (-2°~+2°)	38	3				47	5		
Valgus (≥ 2°)	20	3				9	3		
**Sex**									
Male	50	8	3.771	0.052		30	5	1.024	0.312
Female	187	12				98	9		
**BMI**									
BMI ≤ 25	68	5	0.552	0.458		32	5	0.752	0.386
BMI>25	166	18				96	9		
**Age**									
age ≤ 60	52	5	0.537	0.464		28	3	0.001	0.969
age>60	187	12				100	11		

**Fig. 2:**
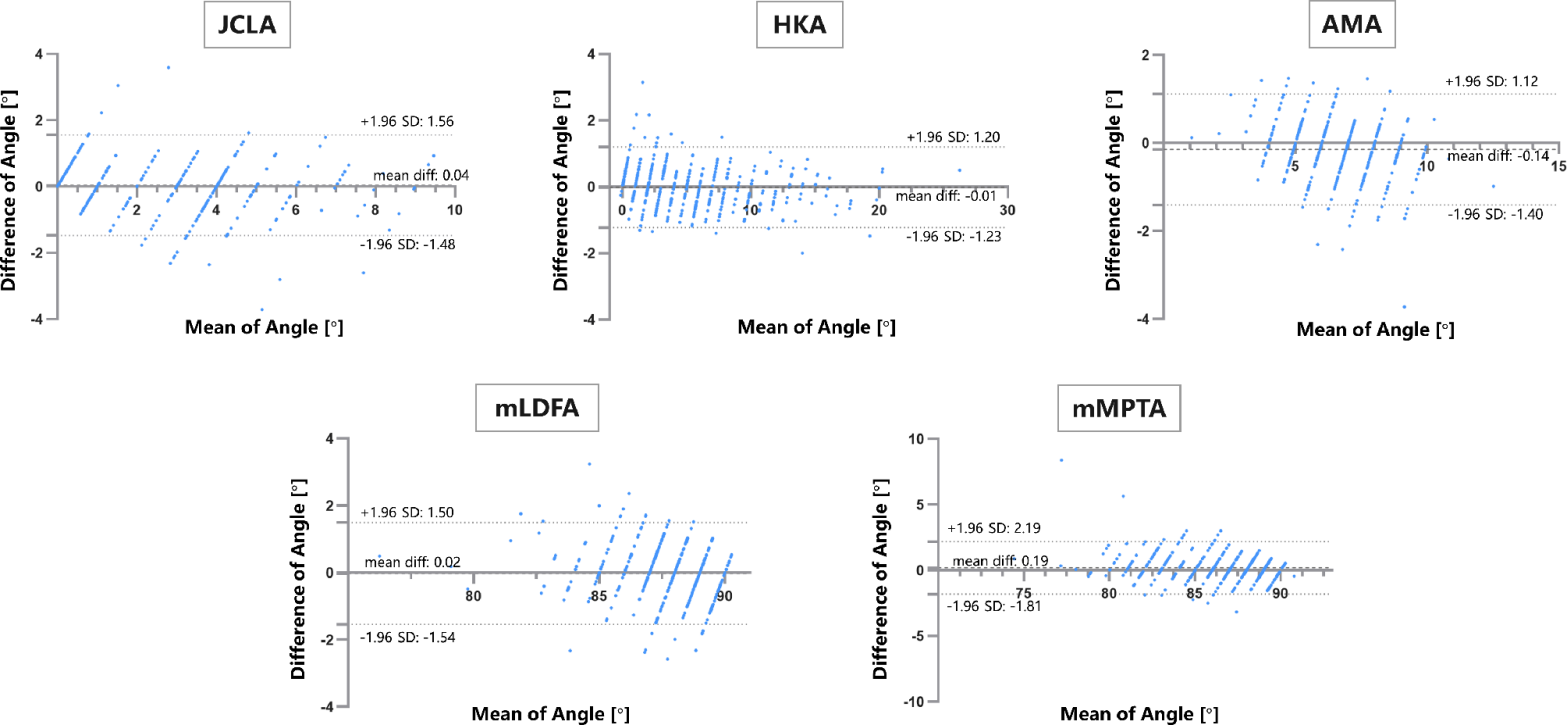
Comparative evaluation of manual and automatic measurement of lower limb alignment. Bland-Altman plots display the agreement between both surgeons’ manual reference and model measurements. diff, difference; SD, standard deviation

The average of manual measurements was considered as ground truth. Bland–Altman plots showed the consistency between prediction angle and ground truth (as shown in Fig. 2). In the Bland–Altman plots, the dashed line denoted the mean of the difference between the two methods that value is -0.014, 0.039, -0.144, 0.187, -0.023 for HKA, JCLA, AMA, mMPTA, mLDFA respectively. And the dotted lines denoted ± 1.96 standard deviations that value was away from the mean of difference. Bland-Altman plots showed that 95% limits of agreement were 1.200 and − 1.227, 1.557 and − 1.479, 1.116 and − 1.404, 2.191 and − 1.814, 1.497 and − 1.54 for HKA, JCLA, AMA, mMPTA, mLDFA respectively.

## Discussion

In this study, for knee OA patients, we presented a fully automated model in portable devices for measuring the alignment of the lower extremity and validated its consistency with surgeons’ measurements. We took five indicators of HKA, JCLA, AMA, mLDFA, mMPTA. The images were taken directly from the portable device without any need for pre-processing. The MRE findings from both the validation and test sets indicate the model’s exceptional generalization and robustness. Additionally, the Bland-Altman analysis demonstrated that the model’s measurements were reliable, whether or not the images had knee prostheses.

According to Table 3, stricter measurement criteria with AD<1° yield lower percentage outcomes for JCLA (79.38%), mMPTA (79.82%), and mLDFA (80.16%), especially for native knee joints. This is primarily because knee OA patients may have varying levels of abrasion and osteophytes present on the tibiofemoral joint surface, causing corresponding landmarks to be dense and indistinct. As a result, there may be a potential discrepancy in anatomical locations (landmarks) detection between manual and model measurements, due to slight variations in practice. As the prosthesis appeared clearly on LLRs, made it easier to identify landmarks during manual measurements. Compared to natural knees, knee joints with prostheses exhibit lower measurement differences (MAD and AD<1°) in JCLA, mMPTA, and mLDFA. Additionally, during the manual examination, surgeons usually zoom in on the femoral condyles and tibial plateau to detect landmarks in images, but the model was able to directly detect all landmarks in the images. Such differences in practice may also affect measurement consistency. Tables 2 and 4 show that the test set patients were more likely to be women, overweight or obese, older, and have lower limb varus deformity. This is consistent with knee OA disease progression and what we have observed in the clinic.

**Fig. 3:**
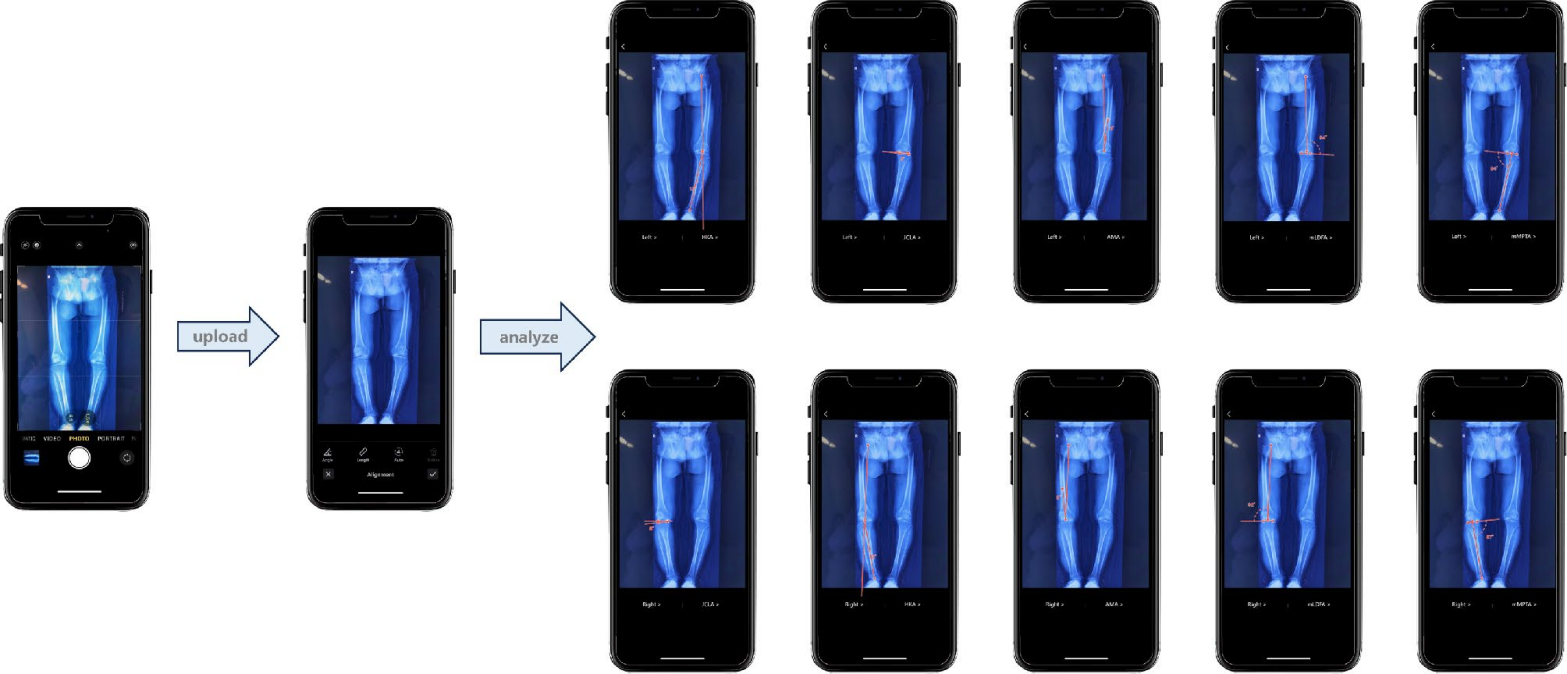
Actual use of the measuring tool schematic in portable device

In research concerning lower limb alignment, multiple measurements are commonly taken by researchers in order to reduce errors [3, 20, 28, 29]. The previous study showed that knee OA patients had a broader distribution of lower limb alignment, making manual measurement more difficult, especially in large patient cohorts for research [27, 28]. In this context, with the development of deep learning, fully automated measurement improves diagnostic accuracy and reproducibility, benefiting related clinical work and research (the actual use of the process is illustrated in Fig. 3). Deep learning is typically used for segmentation, detection, or classification tasks, however, for achieving angle measurements, it is necessary to adapt the complex post-processing of images [30]. In conventional manual measurement techniques, alignments were determined by specifying specific anatomical locations on the limb and then measuring the angle of the connection between the points. Building on this, the proposed model adopted a similar logical approach based on automatic landmark detection and produced accurate and reliable measurement results across a wide range of morphological configurations.

Similar studies have yielded close results for alignment measurements of lower-extremity [3, 20, 30]. Compared to these, we saved more time and achieved automated measurements on portable devices, which greatly facilitates practical use. The HRNet model, proposed for visual recognition problems in 2D images, was adopted in our study [23]. Compared to existing low-resolution classification networks or high-resolution representational learning networks, HRNet was characterised by the following features: it connected high- and low-resolution convolutions in parallel; it maintained high resolution throughout the process, rather than recovering high resolution from low resolution; it repeatedly fused multi-resolution representations, producing rich high-resolution representations with strong positional sensitivity; its storage cost was comparable to available technologies [4]. These features allowed us to guarantee accurate measurements while allowing the model to run on portable devices.

**Fig. 4:**
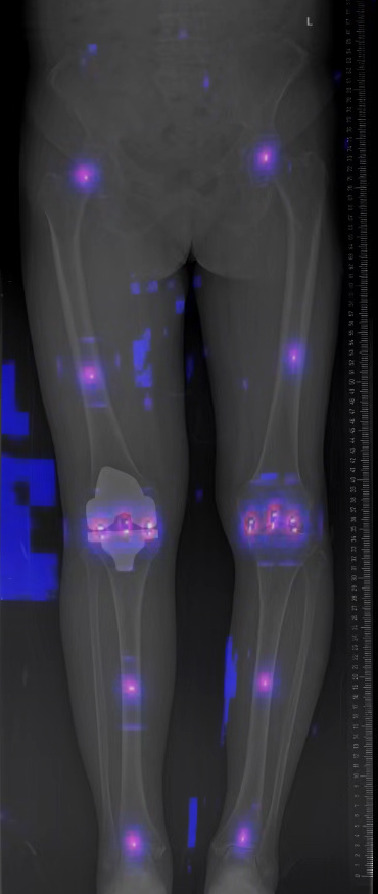
Examples of landmarks detection in the heatmap. LLR was from a 67-year-old female patient diagnosed with knee OA after unilateral TKA and authorized consent was obtained from the patient before the use

This study had limitations. First, unilateral LLRs were excluded for methodological consistency, which limited the diversity of the data to some extent. Second, as knee OA is a chronic degenerative disease, the entire data set was skewed towards middle-aged and older people. The efficacy of this model needs further investigation to assess the fit in younger knee OA patients or non-knee OA patients. Thirdly, knee OA or post-TKA patients were included. Therefore, the accuracy of the model measurements also needed to be further investigated in post-unicompartmental knee arthroplasty or post-internal fixation of the lower limb patients. Fourth, in patients with severe femoral or tibial deformities, traditional alignment assessment methods were not applicable and were not suitable for automated measurement. Fifth, there were still images that could not be identified, requiring further improvements to the model and more explicit requirements for the quality of the LLRs (Fig. 4).

## Conclusion

The deep learning-based measurement tool can be used to assess the lower-limbs alignment of knee OA patients in portable devices. The results are highly reliable, reproducible, and time-saving.

## Data Availability

The datasets used and/or analyzed during the current study are available from the corresponding author upon reasonable request.

## Abbreviations

knee OA: knee osteoarthritis
TKA: total knee arthroplasty
LLR: long-leg radiograph of anteroposterior X-ray
PACS: Picture Archiving and Communication System
HKA: Hip knee ankle angle
JCLA: Joint line convergence angle
AMA: Anatomical mechanical angle
mLDFA: mechanical Lateral distal femoral angle
mMPTA: mechanical Medial proximal tibial angle
MRE: mean radial error
MAD: mean absolute difference
AD: angle deviation
SD: standard deviation
BMI: body mass index
